# *C. trachomatis* pgp3 Antibody Prevalence in Young Women in England, 1993–2010

**DOI:** 10.1371/journal.pone.0072001

**Published:** 2013-08-21

**Authors:** Paddy Horner, Kate Soldan, Sueli M. Vieira, Gillian S. Wills, Sarah C. Woodhall, Richard Pebody, Anthony Nardone, Elaine Stanford, Myra O. McClure

**Affiliations:** 1 School of Social and Community Medicine, University of Bristol, Bristol, United Kingdom; 2 Centre for Infectious Disease Surveillance and Control, Public Health England, London, United Kingdom; 3 Department of Medicine, Imperial College London, London, United Kingdom; 4 Manchester Medical Microbiology Partnership, Public Health England, Manchester, United Kingdom; University of Cambridge, United Kingdom

## Abstract

Seroepidemiology of chlamydia can offer study opportunities and insights into cumulative risk of exposure that may contribute to monitoring the frequency of, and control of, genital chlamydia–the most commonly diagnosed STI in England. We undertook retrospective anonymous population-based cross-sectional surveys using an indirect IgG ELISA for chlamydia Pgp3 antibody. Sera from 4,732 women aged 17–24 years were tested. Samples were taken at 3-yearly intervals between 1993 and 2002, a period during which other data suggest chlamydia transmission may have been increasing, and from each year between 2007 and 2010. Seroprevalence increased in 17–24 year olds over time between 1993 and 2002. Between 2007 and 2010, age-standardised seroprevalence among 17–24 year olds decreased from 20% (95% CI: 17–23) to 15% (95%CI 12–17) (p = 0.0001). The biggest drop was among 20 to 21 year olds, where seroprevalence decreased from 21% in 2007 to 9% in 2010 (p = 0.002). These seroprevalence data reflect some known features of the epidemiology of chlamydia infection, and show that exposure to antibody-inducing chlamydia infection has declined in recent years. This decline was concurrent with increasing rates of screening for asymptomatic chlamydia. Serology should be explored further as a tool for evaluation of chlamydia control, including chlamydia screening programmes.

## Introduction

*Chlamydia trachomatis* is the most common sexually transmitted bacterium in the developed world, with a high prevalence of infection [1]–[3]. In 2012 there were 207,000 diagnoses in England, with the highest rates occurring in young adults [4]. Symptoms of acute infection include painful urination, urethral or vaginal discharge [5], but the majority of infections are asymptomatic. If untreated, chlamydia can give rise to chronic infection and sequelae that include pelvic inflammatory disease, chronic pelvic pain, ectopic pregnancy and tubal factor infertility [5].

Current knowledge of the epidemiology of *C. trachomatis* infection in the UK relies heavily on nucleic acid amplification tests (NAATs) in which *C. trachomatis* DNA is amplified from genital swabs or urine. These specimens tend to be available only from sexually active women. These data provide some information on *current* infection, but information on the prevalence of past infection or the cumulative risk of infection cannot be derived from NAAT testing, nor are the women tested truly representative of the general population [6]–[9]. Until recently, investigation of *C.* *trachomatis* seroepidemiology has been seriously hampered by relatively poor sensitivity and specificity of serological assays [9], [10]. Most chlamydia seroprevalence assays have not been evaluated using sera from patients unambiguously known to be exposed or never exposed to *C. trachomatis* [9], [11] and the older generation assays contained *C. pneumoniae* homologues, a common respiratory pathogen. As a result, chlamydia serology as a means of understanding the natural history of infection fell out of favour [11]. In 2009, we produced an “in-house” indirect immunoglobulin G (IgG) enzyme-linked immunosorbent assay (ELISA) based on the *C. trachomatis*-specific antigen Pgp3 and demonstrated its potential for utilisation in seroepidemiological surveys [10]. We evaluated this ELISA against three commercial MOMP peptide ELISA assays, using sera from 164 women who had been infected with *C. trachomatis* and from 722 chlamydia-negative children aged 2–13 years [10]. This ELISA was significantly more sensitive than the best performing MOMP peptide ELISA (Anilabsystems) (73.8% vs 59.8% *P* = 0.001) (95% CI 66.5% to 79.9%) and had a specificity of 97.6% (95% CI 96.2% to 98.6%) amongst 164 women (at least 14% better than the best assays available commercially [10]), with no evidence of detection of cross-reactive *C. pneumoniae* antibody in a study of 722 samples from children [10].

There are challenges to the interpretation of chlamydia seroprevalence. Seroprevalence is likely to underestimate the proportion of women who have had a chlamydia infection as only 68% of infected women remain antibody-positive after 6 months [12], unless they are subsequently re-infected, and infection in the upper genital tract is more likely to induce an antibody response than a mucosal infection at the cervix alone [13]. There may also be differences in the rate of antibody inducing infection depending on factors such as whether or not there is treatment and how soon this is given post-infection [14].

Data from surveillance of sexually transmitted infections (STIs) diagnosed in GUM clinics in England have suggested there was a fall in transmission of STIs in 1986–7 associated with the campaigns for safer sex designed to prevent HIV transmission. There were falls in rates of gonorrhoea and genital herpes and an interruption to rises in genital warts, and an overall reduction in new GUM clinic attendances requiring treatment (whilst attendances not requiring treatment increased). Then, after this period of reduction, STI rates overall, and notably those for gonorrhoea, syphilis and viral STIs, began to increase from 1995 [15]. Increases in sexual risk behaviours have also been described [16]. Hence, we would expect there to have been increased exposure to *C. trachomatis* in women becoming sexually-active (i.e. reaching around 17–18 years of age) from 1993 onwards. Screening of asymptomatic individuals for chlamydia has increased markedly in recent years, particularly since the National Chlamydia Screening Programme (NCSP) began to offer sexually active people under 25 years screening annually and on change of sexual partner in order to detect infections in young people and reduce the prevalence of infection and incidence of sequelae [17]–[19]. In 2007/08, around 300,000 tests were conducted via the NCSP, increasing to 1.4 million by 2010/11. Tests in all settings (including Genitourinary medicine (GUM) clinics) in 2010/11 summed to over 2.1 million, with approximately 43% of young women and 22% of young men tested (on the assumption that each test represents an individual) [20]. The impact of widespread, opportunistic screening, as practised in England, on chlamydia epidemiology has not been empirically established.

We report the results of the first population-based study of antibodies to the *C. trachomatis*-specific antigen Pgp3 in England using the recently developed and validated Pgp3 ELISA and a population-based serum collection from 17–24 year old women. This assay performs significantly better in women than men with a sensitivity of 44.2% in latter [10]. We explore two expected epidemiological variations: increased (cumulative) infection with age, up to 25 years, and the increased infection rates over time from the mid-1990s to early 2000s that have been described by others [15]. We then explore seroprevalence changes in more recent years.

## Methods

Serum specimens were obtained from Public Health England’s Sero-Epidemiology Unit collection. This consists of unlinked residual sera submitted to laboratories in England for routine microbiological or biochemical investigations. Sera from individuals known to be immuno-compromised and repeat sera from the same individuals were excluded [21], [22]. In addition, we excluded any sera known to have been collected at a GUM clinic. A small number of selected specimens (0.3%) were of insufficient volume for testing.

Sera from 4,732 women aged 17–24 years came from twenty-nine laboratories in eight regions of England that had collected samples between 1993 and 2010. The sample was chosen pragmatically, with numbers limited by available sera and competing needs for use of these sera. Between 1993 and 2002 samples were taken at 3-yearly intervals spanning the years of putative increases in chlamydia transmission. The choice of every 3^rd^ year was judged sufficient to capture trends over time and also captured the aging of 2-year birth cohorts. We did not sample from 2003–2006 because collected sera numbers were low during the mid-2000s. From the more recent years, 2007 to 2010, samples were taken from each year to maximise data for trend analyses over this shorter period. The age-groups 17–18, 20–21 and 23–24 were sampled from every collection year to enable analysis by these age groups across the whole period. From 2007 onwards, 19 and 22 year olds were also sampled to give a full range by year of age for this recent period. As seroprevelance is lower at younger ages (less cumulative incidence) under 17 year olds were not included as testing these ages was considered less efficient in generating information about trends over time, given seroprevalence is a tool for cumulative infection, not incident infection (in contrast to NAAT data). This sampling frame and the numbers by year and age were determined as likely to maximise the precision of trends by age and time up to 2002 and of age-standardised prevalence for 17–24 year olds from 2007 to 2010, given the numbers of stored sera available.

Samples were tested for *C. trachomatis* Pgp3 antibody at Imperial College London using the indirect Pgp3 assay described previously [10]. Briefly, a Maxisorp 96-well microtitration plate (Nunc, DK) was coated with 100 ng/well of the Pgp3 protein and blocked with 1% Hammersten casein in PBS-Tween-20 (blocking buffer). Antisera were assayed at a dilution of 1∶100 in blocking buffer, secondary antibody added, then TMB substrate. The reaction was allowed to develop and absorbance read at 450 nm. Sera were considered to be seropositive at the 0.473 cut-off, determined using ROC curve analysis with 356 *C. trachomatis*-positive sera and 722 paediatric (*C. trachomatis-negative*) sera [10]. We repeated the assay on a random selection of 80 *C. trachomatis* Pgp3 antibody-negative paediatric control sera which were assayed in duplicate and the mean determined. The mean absorbance value for this assay and the original were compared by Student’s t-test and were not significantly different (p>0.05).

### Statistical Analyses

Crude seroprevalence was calculated by age group and year. Age-standardised seroprevalence was calculated for the combined age group data using direct standardisation. The population of England in 2004 constituted the reference population [23]. Analyses for trends by increasing age were conducted within birth cohorts, to reduce confounding by secular trends. Trends over time or by birth cohort were examined using the score test for trend. Analyses for trend over time were conducted separately for data from 1993 to 2002 and for data from 2007 to 2010. All analyses were carried out using STATA 12.0 (StataCorp. 2011. Stata Statistical Software: Release 12. College Station, TX: StataCorp LP).

The data are held by Public Health England and may be obtained by contacting the author for correspondence.

### Ethics Statement

National Research Ethics Service (NRES) approval was granted by the Joint University College London/University College London Hospital (UCL/UCLH) Committees on the Ethics of Human Research. (Research Ethics Committee number 05/Q0505/45).

## Results

We tested 1,314 women aged 17–18 years, 1,415 aged 20–21, 1,420 aged 23–24 and 583 aged 19 or 22 at the time their blood sample was taken. Of the total 4,732 serum samples tested in our indirect Pgp3 ELISA, 826 of them were antibody positive (17.5%).

### Changes in Pgp3 Seroprevalence with Age within Birth Cohorts

**Figure 1:**
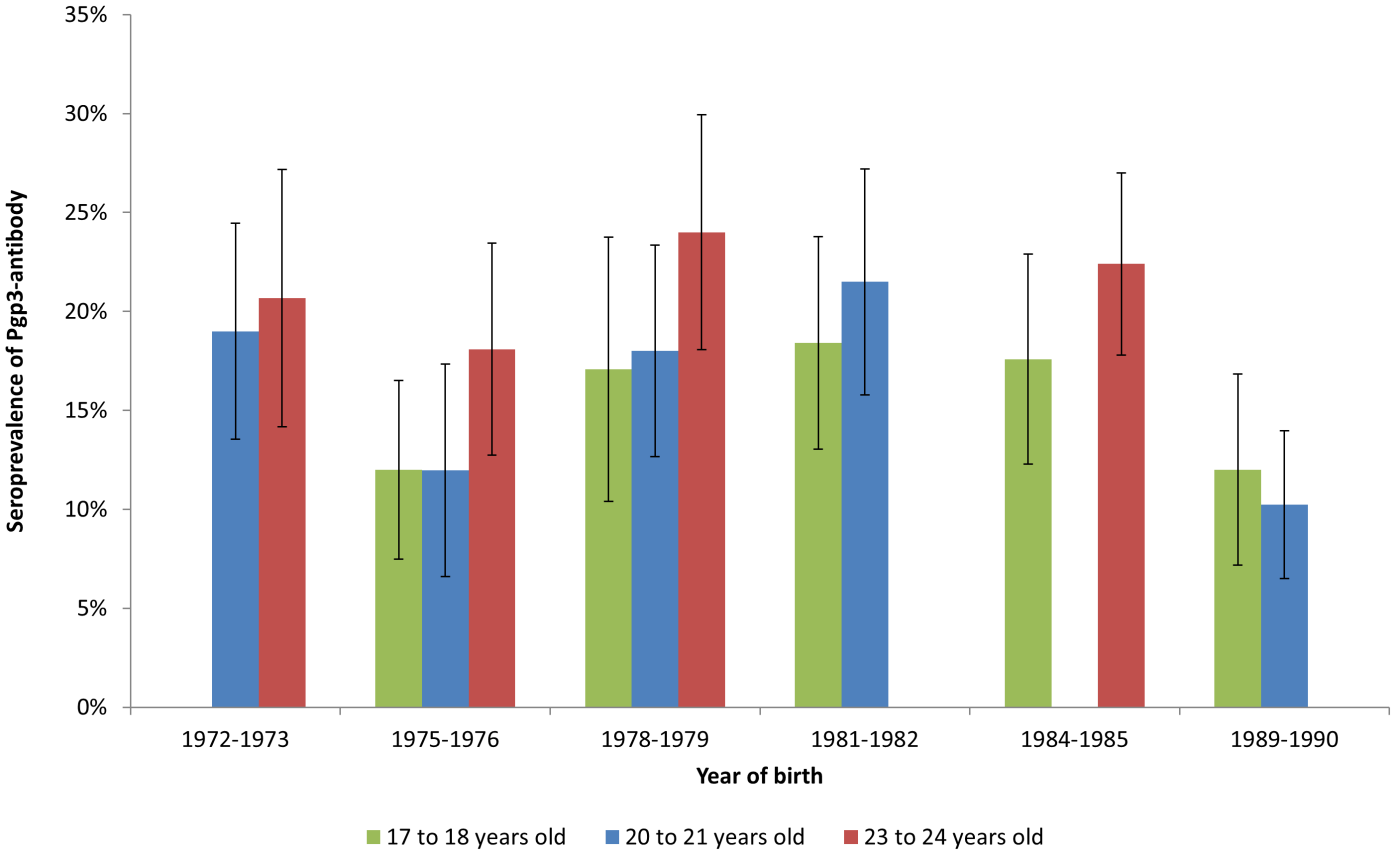
Cumulative *C. trachomatis* seroprevalence by birth cohort within three age groups. Missing bars occur where years of birth were not represented in the samples (selected by age and year of collection).

Increasing seroprevalence with age was seen: point estimates for crude seroprevalence were higher in the older age groups within each two-year birth cohort, as would be expected for a marker of cumulative infection, apart from those born between 1989 and 1990 (Figure 1). However, the increases by age, from 17–24 years, were small and trends by age within birth cohorts were not statistically significant.

### Changes in Pgp3 Seroprevalence between 1993 and 2002

**Figure 2:**
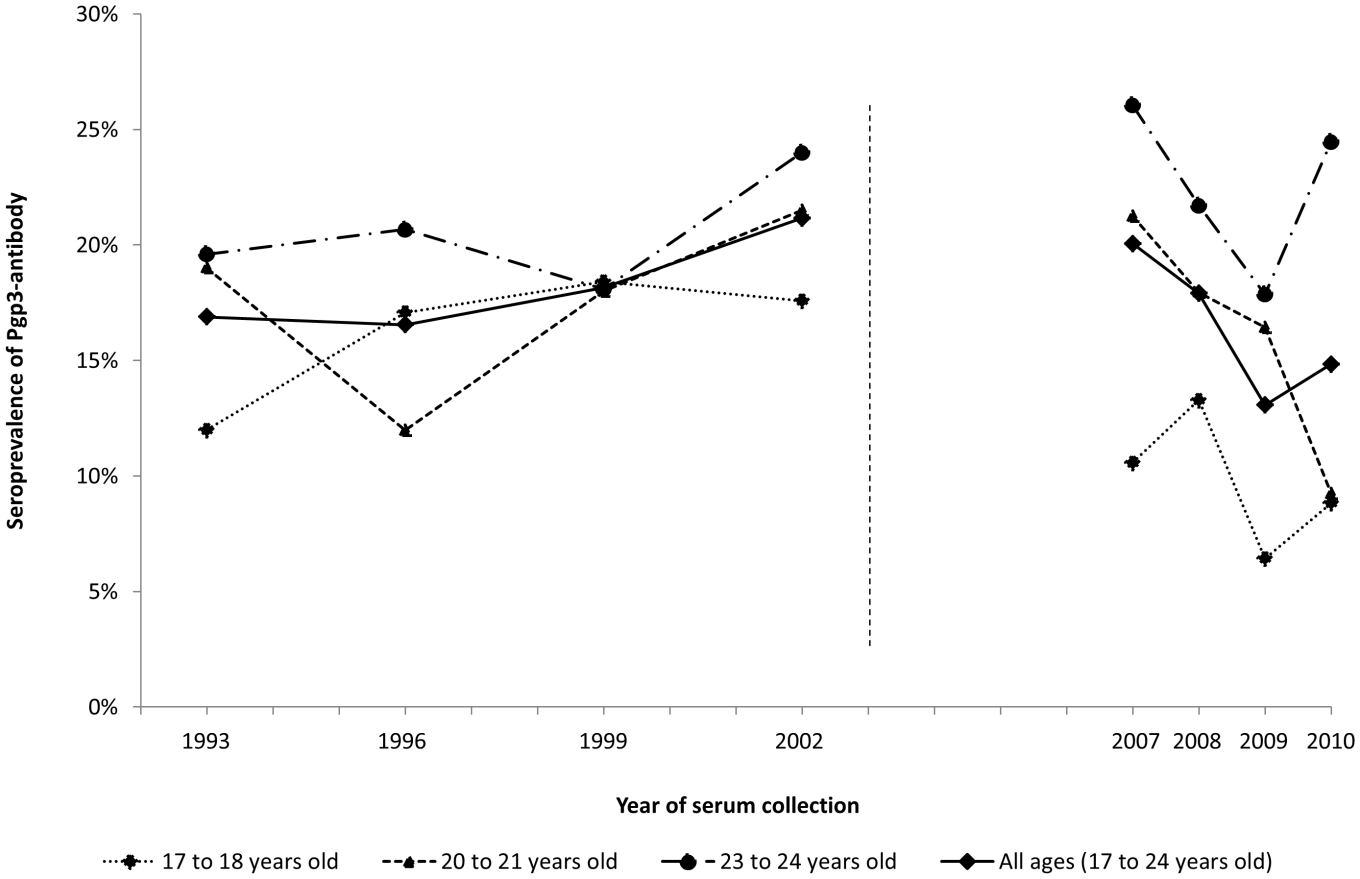
Seroprevalence of *C. trachomatis* antibody by age group and year of sample collection. (For continuity of data in this table, and because they are not very meaningful as single-year age groups, we do not show the data for 19 and 22 year olds).

Between 1993 and 2002, age-standardised seroprevalence increased from 17% to 21% (p = 0.053) (Table 1 and Figure 2). Seroprevalence increase over time during this period was seen in all age groups, although none was statistically significant. Among women turning 17 or 18 between 1993 and 1999 (i.e. born 1975–82), age-specific seroprevalence increased over time (Figure 1). Seroprevalence at age 17–18 increased from 12% in 1993 to 17% in 1996, and to 18% in 1999 (p = 0.078). Seroprevalence at age 20–21 increased from 12% in 1996 to 18% in 1999 and to 22% in 2002 (p = 0.025), while seroprevalence at age 23–24 increased from 18% in 1999 to 24% in 2002 (p = 0.15).

**Table 1:** Seroprevalence of *C. trachomatis* antibody by age group and year of sample collection.

Age group (years)	Year of collection	Tested	Positive	Prevalence (%)	95% CI		P-value for trend^a^
17–18	1993	200	24	12	7	−17	*1993–2002*
	1996	123	21	17	10	−24	
	1999	201	37	18	13	−24	0.110
	2002	199	35	18	12	−23	
	2007	104	11	11	5	−17	*2007–2010*
	2008	143	19	13	8	−19	
	2009	140	9	6	2	−11	0.268
	2010	204	18	9	5	−13	
20–21	1993	200	38	19	14	−24	*1993–2002*
	1996	142	17	12	7	−17	
	1999	200	36	18	13	−23	0.330
	2002	200	43	22	16	−27	
	2007	193	41	21	15	−27	*2007–2010*
	2008	156	28	18	12	−24	
	2009	140	23	16	10	−23	**0.002**
	2010	184	17	9	5	−13	
23–24	1993	199	39	20	14	−25	*1993–2002*
	1996	150	31	21	14	−27	
	1999	199	36	18	13	−23	0.402
	2002	200	48	24	18	−30	
	2007	192	50	26	20	−32	*2007–2010*
	2008	152	33	22	15	−28	
	2009	140	25	18	11	−24	0.572
	2010	188	46	24	18	−31	
Age groups combinedb,c	1993	599	101	17	14	−20	*1993–2002*
	1996	415	69	17	13	−20	
	1999	600	109	18	15	−21	0.053
	2002	599	126	21	18	−24	
	2007	489	102	19	16	−23	*2007–2010*
	2008	451	80	18	14	−21	
	2009	420	57	14	10	−17	**0.001**
	2010	576	81	14	12	−17	
All years of age, 17 to 24^c^	2007	635	135	20	17	−23	*2007–2010*
	2008	618	111	18	15	−21	
	2009	560	73	13	10	−16	**0.0001**
	2010	706	102	15	12	−17	

a Score test for trend.

b 17–18 year olds, 20–21 year olds and 23–24 year olds combined. For continuity of data in this table, and because they are not very meaningful as single-year age groups, we do not show the data for 19 and 22 year olds.

c Prevalence age standardised to ONS 2004 population.

The pattern of seroprevalence by age and over time between 1993 and 2002 was, therefore, broadly reflecting the epidemiological variations we expected to see.

### Changes in Pgp3 Seroprevalence from 2007–2010

For the more recent period, between 2007 and 2010, age-standardised seroprevalence among the combined age groups (17–24 years) decreased from 20% (95% CI 17%–23%) to 15% (95%CI 12%–17%) (p = 0.0001) (Table 1). The biggest fall was observed among 20 to 21 year olds, where seroprevalence more than halved from 21% in 2007 to 9% in 2010 (p = 0.002) (Table 1 and Figure 2). The decline among 17–18 year olds was smaller (from 11% to 9%) and not statistically significant (p = 0.27). There was no consistent trend in seroprevalence among 23–24 year olds between 2007 and 2010.

## Discussion

Based on our sample, chlamydia Pgp3 antibody prevalence increased non-significantly, but consistently, by birth cohort in women reaching 17 between 1993 and 2000, in 17–24 year olds over time between 1993 and 2002, and within birth cohorts by age (with the exception of those born 1972–1973). This is consistent with expectations based on other epidemiological information. Chlamydia seroprevalence then declined steadily in young women under 24 years of age between 2007 and 2010, the greatest reduction being observed in 20–21 year olds. Our findings suggest that exposure to infection, or at least to antibody-inducing infection, may have declined in recent years.

The population tested in the survey consists of individuals accessing healthcare in England with collection of blood samples for diagnostic or screening purposes. Previous studies using the same collection have found the sample source is broadly representative of the general population, at least for relatively common infections. Although we can not know whether individuals tested in this study are of higher or lower risk of STIs than the general population, this sample source has been used informatively for another STI, namely HPV [24]. Neither do we have information on changes in selection bias in our sample source over time. The study was restricted to, and somewhat limited by, the available numbers of sera by age and year. The Pgp3 antibody ELISA sensitivity and specificity has been rigorously defined using characterised sera from 164 women known to have been infected and 722 chlamydia-negative children aged 2–13 years [10]. We have also extended this analysis and observed that Pgp3 antibody decreases in the first 6 months following infection then plateaus and that chlamydia antibody is more readily detectable in women with multiple episodes of infection [12]. Prospective population-based studies would be desirable to enable more accurate characterisation of how Pgp3 antibody changes with time since infection.

The seroprevalence at age 17–18 was high. A nationally-representative survey of sexual behaviour in 2000 found that 50% of women reported sexual debut by age 17 [25]. The median age of first sexual intercourse reported by the Health Survey for England in 2010 was 17 [26]. We would need to consider partner change rates and chlamydia transmission rates, as well as likelihood of very recent infection and therefore higher seroprevalence (before any waning) to investigate whether the level of seroprevalence we found is fully consistent with knowledge about sexual exposure to chlamydia. The seroprevalence unfailingly exceeded the proportion of women found to be infected by NAATs in other studies [1], [3] and of women who had undergone chlamydia screening in recent years [4], as expected for a cumulative and specific marker of infection. This relative magnitude is as we would expect. Unlike most surveys of chlamydia infection that require genital samples, our population was not restricted to sexually active women.

The lack of a strong and steady increase in seroprevalence by age from 17 to 24 years within birth cohorts suggests that seroprevalence is not an absolute marker of all past chlamydia infection. Such a marker would be expected to rise with increasing numbers of sexual partners in this group. However, there are other possible explanations for this. The population tested in the survey may differ by age in terms of how well they represent their age cohort with respect to chlamydia infection. Reasons for blood testing do vary by age and some of these reasons may be associated with sexual behaviour *e.g*. antenatal testing. As this was an anonymous study, we were unable to explore these selection biases further, neither were we able to explore the relationship between seropositivity, symptoms and treatment among the women tested. Another possible anomaly was a large drop in seroprevelance amongst 17–18 year olds between 2002 and 2007. This may simply be a sample size issue as the confidence limits overlap.

Women aged 20–21 in 1993 had a high seroprevalence, relative to this age in later years. These women would have turned 17 in 1990 and earlier. This suggests that chlamydia infection in young people continued to fall through the late 1980s and early 1990s, possibly as a result of safer sexual practices [15], [27]. There has been only one previous study looking at chlamydia seroprevalence in a population based study in Europe; Lyytikainen *et al.* studied *C. trachomatis* seroprevalence in a subcohort of 8000 women stratified by calendar years (1983–1989, 1990–1996, 1997–2003) and age at time of sample, from a Finnish population serum bank. Participants were women under 29 yrs, having at least two pregnancies [28]. The study assessed prevalence of past exposure to genital *C. trachomatis*, using Anilabsystems MOMP peptide ELISA, and explored changes over time by comparing age-specific prevalence at different time-points. Unlike our observations, a declining prevalence of *C. trachomatis* antibody was observed from 1983 to1989 and 1997–2003 [28]. The authors postulated that this may be due to more intensive testing and possible earlier diagnosis, resulting in poorer humoral responses, i.e. fewer antibody-inducing infections in these women. However, bias in the sample selected (2 pregnancies required for inclusion), uncertainties with the methodological analysis and changes in fertility patterns over the 20 years of the study which may have altered the characteristics of those having children under the age of 30 [29] may also have contributed. In addition, the Anilabsystems MOMP peptide ELISA is less sensitive than the pgp3 ELISA and repeat infection does not result in a sustained greater persistence of antibody compared to a single infection [10], [12]. If age at first, and only, infection had decreased, we might expect a reduction in age-specific seroprevalence, as seroprevalence decreases with time since infection [12]. This seems an unlikely explanation of our findings, as earlier age of sexual activity is more likely to be associated with an ongoing higher risk of infection.

The decline in seroprevalence during the recent period, 2007–2010 is striking (Table 1 and Figure 2). During this time period, substantial efforts have been directed to increase screening of young sexually active people for chlamydia, resulting in an increase from 1.3 million tests in 2008 to 2.1 million tests in 2011 [20]. Screening seeks to reduce the number of infections, progression to disease and duration of infection and one would, therefore, expect seroprevalence to fall if screening was effective, whatever the balance of incidence, re-infection and frequency and timing of treatment in determining seroprevalence. For example, if treatment prevents the development of antibody, seroprevalence could fall without screening necessarily being effective at reducing transmission in the population. Mathematical modelling suggests that the levels of screening uptake achieved in England should have reduced population prevalence [30]. Other aspects of the National Sexual Health Strategy introduced in England in 2001, which also encouraged safer sexual practices, including condom usage and rapid access to treatment for symptomatic individuals, may also have been influential [18]. Although the likelihood of seropositivity is not equal for all infections [12]–[14] the extent to which seroprevalence is a measure of antibody-inducing infection may make it a more interesting observation in relation to chlamydia control than a measure of prevalence alone, particularly if early treatment of asymptomatic infection reduces both seroconversion and risk of chronic sequelae [14], [31].

Seroprevalence for Pgp3 offers an additional viewpoint on the epidemiology of chlamydia in England, and a rare view of the risk of chlamydia infection (at least, antibody-inducing infection) in female subjects who are not indirectly selected via specimen type for their known sexual activity. Our data suggest that there is already a substantial risk of chlamydia infection in girls in England by the age of 17, which supports the introduction of annual screening of sexually active girls at a young age.

This study demonstrates that seroprevalence has the potential to be an important new tool for improving our understanding of chlamydia epidemiology and to contribute to evaluation of chlamydia control. One advantage of this tool is it enables the use of opportunistic collections of sera that do not suffer from the costs and participation biases typical of studies requiring collection of genital samples for DNA testing. Screening of asymptomatic young people is recommended in a number of other countries, including the USA (sexually active young females) [32] and evaluation tools are clearly needed to measuring the impact of this activity [19], [32].

The likelihood that diagnosis (and treatment) by screening reduces serocoversion, and that antibody levels wane over time and are boosted by repeat infections are complications to the use of these data to estimate age-specific infection rates, increasingly so with increasing age. Analyses of antibody titres may give some insight into these aspects, and are one area for further work. We have observed that Pgp3 antibody decreases in the first six months following infection then reaches a plateau and that chlamydia antibody is more often detected following multiple episodes of infection [12]. Further investigation of the natural history of antibody responses is, therefore, warranted, ideally via prospective studies that can show changes with time since infection. Serology should be explored further as a tool for evaluation of chlamydia control, including chlamydia screening programmes.
